# Varicella Pneumonia Complicating Pregnancy: A Report of Seven Cases

**DOI:** 10.1155/S1064744996000683

**Published:** 1996

**Authors:** Teresa J. Schutte, Louise C. Rogers, Pleas R. Copas

**Affiliations:** Department of Obstetrics and Gynecology University of Tennessee Medical Center 1924 Alcoa Highway Box U-28 Knoxville TN 37920 United Kingdom

## Abstract

*Background:* Pneumonia is the most common complication of varicella-zoster infection in adults and has potentially devastating effects when complicating pregnancy. Due to the significant morbidity and mortality associated with this complication during pregnancy and the small number of reported cases in the literature, we present this report to help educate physicians who care for pregnant women.

*Cases:* Seven patients are presented in this report. These patients presented at various stages in pregnancy, from 17 to 31 weeks of gestation. Three of the patients had unremarkable hospital courses. Three of the patients had hospital stays over 21 days in duration. One patient died from complications of varicella pneumonia after 31 days of hospitalization. The obstetric outcomes of the 7 patients described include 1 non-viable delivery at 20 weeks gestation, 3 term deliveries, 2 preterm deliveries, and 1 patient who has not yet delivered. All of the patients presented were treated with intravenous acyclovir therapy. Of the patients described, 3 required intubation and ventilatory support. Other complications encountered include disseminated intravascular coagulation (DIC), adult respiratory distress syndrome (ARDS), metabolic encephalopathy, pneumothorax, superimposed bacterial pneumonia, and sepsis.

*Conclusion:* The course and treatment of varicella pneumonia complicating pregnancy are discussed. Current recommendations regarding the use of varicella-zoster immune globulin (VZIG) are also reviewed.


Infectious Diseases in Obstetrics and Gynecology 4:338–346 (1996)
© 1997 Wiley-Liss, Inc.

### Varicella Pneumonia Complicating Pregnancy: A Report of Seven Cases

**Teresa J. Schutte, Louise C. Rogers, and Pleas R. Copas***
*Department of Obstetrics and Gynecology, University of Tennessee Medical Center, Knoxville, TN*

### Abstract

***Background:*** **Pneumonia is the most common complication of varicella-zoster infection in adults and has potentially devastating effects when complicating pregnancy. Due to the significant morbidity and mortality associated with this complication during pregnancy and the small number of reported cases in the literature, we present this report to help educate physicians who care for pregnant women.**

***Cases:*** **Seven patients are presented in this report. These patients presented at various stages in pregnancy, from 17 to 31 weeks of gestation. Three of the patients had unremarkable hospital courses. Three of the patients had hospital stays over 21 days in duration. One patient died from complications of varicella pneumonia after 31 days of hospitalization. The obstetric outcomes of the 7 patients described include 1 non-viable delivery at 20 weeks gestation, 3 term deliveries, 2 preterm deliveries, and 1 patient who has not yet delivered. All of the patients presented were treated with intravenous acyclovir therapy. Of the patients described, 3 required intubation and ventilatory support. Other complications encountered include disseminated intravascular coagulation (DIC), adult respiratory distress syndrome (ARDS), metabolic encephalopathy, pneumothorax, superimposed bacterial pneumonia, and sepsis.**

***Conclusion:*** **The course and treatment of varicella pneumonia complicating pregnancy are discussed. Current recommendations regarding the use of varicella-zoster immune globulin (VZIG) are also reviewed. Infect. Dis. Obstet. Gynecol. 4:338–346, 1996.** © 1997 Wiley-Liss, Inc.

Key words
acyclovir; varicella-zoster immune globulin; varicella vaccine, pneumonitis

Primary varicella-zoster infection in pregnancy is estimated to occur in 1–5/10,000 pregnancies.[1,2] Though commonly a mild infection in children, varicella-zoster infection in adults is associated with higher morbidity and mortality. The most common complication of varicella infection in adults is pneumonia. Approximately 16% of adults with varicella infection will demonstrate evidence of pulmonary involvement.[2] Although the incidence of varicella pneumonia does not appear to be increased in pregnancy, it is well established that the morbidity and mortality from this infection in pregnancy are higher. Mortality from varicella pneumonia complicating pregnancy is reported to be as high as 41%.[3] In our institution over the past 5 years, we have observed 7 cases of varicella pneumonia complicating pregnancy, which we now present with a discussion of the pertinent literature. Table 1 presents a summary of the salient features of each case and Table 2 presents initial laboratory data.

### CASE REPORTS

### Case 1

C.H. was a 35-year-old white female gravida 2, para 1, with an intrauterine pregnancy at 25 weeks ges-

*Correspondence to: Dr. Pleas R. Copas, Department of Obstetrics and Gynecology, University of Tennessee Medical Center, 1924 Alcoa Highway, Box U-28, Knoxville, TN 37920.

Received 9 July 1996
Accepted 14 January 1997

TABLE 1. Patient and case characteristics[a]

| Case no. | Gestational age at presentation (weeks) | Length of hospital stay (days) | Acyclovir dose (mg/kg) | Duration of IV acyclovir therapy in days (days of oral dosing | Antibiotics used (dosages—IV unless otherwise specified) | Mechanical ventilation required | VZIG use | Fetal outcome | Maternal complications |
|---|---|---|---|---|---|---|---|---|---|
| 1 | 25–26 | 10 | 12.0 | 9 (0) | None | No | No | Term SVD, normal male | None |
| 2 | 17–18 | 32 | 12.4 | 8 (0) | Erythromycin (1 g q 6° × 5 days) Vancomycin (1.25 g q 12° × 16 days) Pipericillin (4 g q 4° × 7 days) Tobramycin (110 mg q 12° × 5 days Ceftazidime (2g q 8° × 8 days) | Yes | No | Term SVD, normal male | ARDS, seizure disorder, superimposed bacterial pneumonia, staphylococcal septicemia, metabolic encephalopathy, cholelithiasis, sinusitis |
| 3 | 20–21 | 31 | 6.5 increased to 10.0 | 10 (0) | Erythromycin (1 g q 6° × day) | Yes | No | SVD of non-viable male (normal) | Maternal death, DIC, ascites, pneumothorax, superimposed candidal pneumonitis |
| 4 | 30–31 | 22 | 12.5 | 7 (0) | Cefuroxime (1.0 g q 8° × 8 days) Vancomycin (1.25 g q 12° × 10 days) Tobramycin (100 mg q 12° × 10 days) Ceftazidime (1.0 g q 8° × 10 days) | Yes | Yes | PTD by cesarean section, normal female | ARDS, staphylococcal septicemia |
| 5 | 27–28 | 21 | 5.0 | 5 (5) | Ceftriaxone (2 g q 8° × 3 days) Clarithromycin (500 mg po bid × 9 days) | No | No | PTD by cesarean section, normal male | None |
| 6 | 30–31 | 3 | 10.3 | 3 (5) | None | No | No | Term SVD, normal female | None |
| 7 | 23–24 | 5 | 10.0 | 5 (5) | Cefazolin (2 g q 8° × 4 days) | No | No | Term SVD, normal female | Subsequent bacterial pneumonia requiring 2-day hospitalization in third trimester |

[a]IV, intravenous; SVD, spontaneous vaginal delivery; PTD, preterm delivery; ARDS, adult respiratory distress syndrome; DIC, disseminated intravascular coagulation.

TABLE 2. Descriptive laboratory data[a]

| Case No. | Hgb (mg/dl) | WBC | % Segs | % Bands | Arterial pH | $pO_2$ | $pCO_2$ | $S_AO_2$ | Varicella serology |
|---|---|---|---|---|---|---|---|---|---|
| 1 | 10.6 | 10,900 | 50 | 24 | 7.448 | 68 | 30 | 95 | Unavailable |
| 2 | 13.0 | 5,700 | 70 | 0 | 7.419 | 40 | 33 | 76 | Positive |
| 3 | 11.6 | 13,900 | 85 | 0 | 7.446 | 50 | 32 | 87 | Positive |
| 4 | 11.0 | 5,400 | 83 | 30 | 7.495 | 84 | 28 | 97 | Unavailable |
| 5 | 12.8 | 4,000 | 73 | 7 | 7.405 | 80 | 34 | 96 | Negative |
| 6 | 12.4 | 8,400 | 91 | 0 | 7.476 | 72 | 31 | 96 | Negative |
| 7 | 11.0 | 7,500 | 65 | 15 | 7.481 | 61 | 25 | 93 | Positive |

[a]Hgb, hemoblobin; Segs, segmented neutrophils.

tation who presented with a 3-day history of lethargy, pruritic vesicular rash (involving the trunk, face, and extremities), non-productive cough, and progressively worsening shortness of breath. Her past medical history was significant for exposure to a child with documented varicella infection 2 weeks prior to initial presentation and tobacco use. Initial physical examination revealed tachycardia, tachypnea, a temperature of 36.7°C, scattered rhonchi bilaterally on auscultation of the lungs, and a diffuse vesicular rash involving the skin and oropharynx. The admission chest X-ray revealed diffuse bilateral air space densities consistent with varicella pneumonia. An obstetric ultrasound on presentation revealed a viable fetus with no evidence of fetal anomalies. The patient was started on intravenous acyclovir, oxygen ($FiO_20.50$) by ventimask, intermittent positive pressure breathing, and aggressive pulmonary toilet. All viral, bacterial, and mycoplasma cultures were negative. She improved, was discharged, and returned at 41 weeks gestation to have an uncomplicated spontaneous vaginal delivery of a normal male infant weighing 3,039 g.

### Case 2

Y.B. was a 21-year-old white female gravida 4, para 2, who presented at 17 weeks gestation with a 4-day history of cough, rash, malaise, fever, emesis, and shortness of breath. Her past medical history was significant for tobacco use, varicella infection at age 5 years, and recent exposure to a child with varicella-zoster infection. Physical examination revealed a mild tachycardia, tachypnea, a temperature of 39.2°C, fine crackles bilaterally on auscultation of the lungs, and vesicular rash present over the face, chest, back, and oropharynx. The initial chest X-ray revealed a severe, patchy, and diffuse alveolar infiltrate (Fig. 1). She was admitted with a diagnosis of varicella pneumonia and started on acyclovir. She was intubated on the 2nd hospital day and required prolonged ventilatory support. Her hospital course was complicated by adult respiratory distress syndrome (ARDS), tonic-clonic seizures requiring intravenous phenytoin, superimposed bacterial pneumonia, staphylococcal septicemia, metabolic encephalopathy, thrombocytopenia, anemia, cholelithiasis, sinusitis, elevated liver enzymes, requirement for enteral nutrition, and anemia requiring transfusion of 6 units of packed red blood cells. An obstetric ultrasound revealed a viable intrauterine pregnancy with no evidence of fetal anomalies. The remainder of her prenatal care was uncomplicated. She was discharged and returned at 41 weeks gestation to have a spontaneous vaginal delivery of a normal male infant weighing 2,869 g.

### Case 3

T.M. was a 19-year-old white female gravida 2, para 1, who presented at 20 weeks gestation with a 2-day history of malaise, nausea, emesis, fever, mild abdominal pain, and diffuse vesicular rash. The patient denied any pulmonary symptoms. Her past medical history was unremarkable with the exception of exposure to varicella-zoster infection 2 weeks prior to the onset of her symptoms. Initial physical examination revealed tachycardia, mild tachypnea, a temperature of 37.8°C, and diffuse vesicular lesions consistent with varicella in various stages of eruption scattered over her face, trunk, and extremities. Auscultation of the lungs revealed normal findings without adventitious lung sounds. Within 24 h of admission, the patient developed symptoms of cough, shortness of breath, and a temperature of 38.6°C. A chest X-ray was consistent

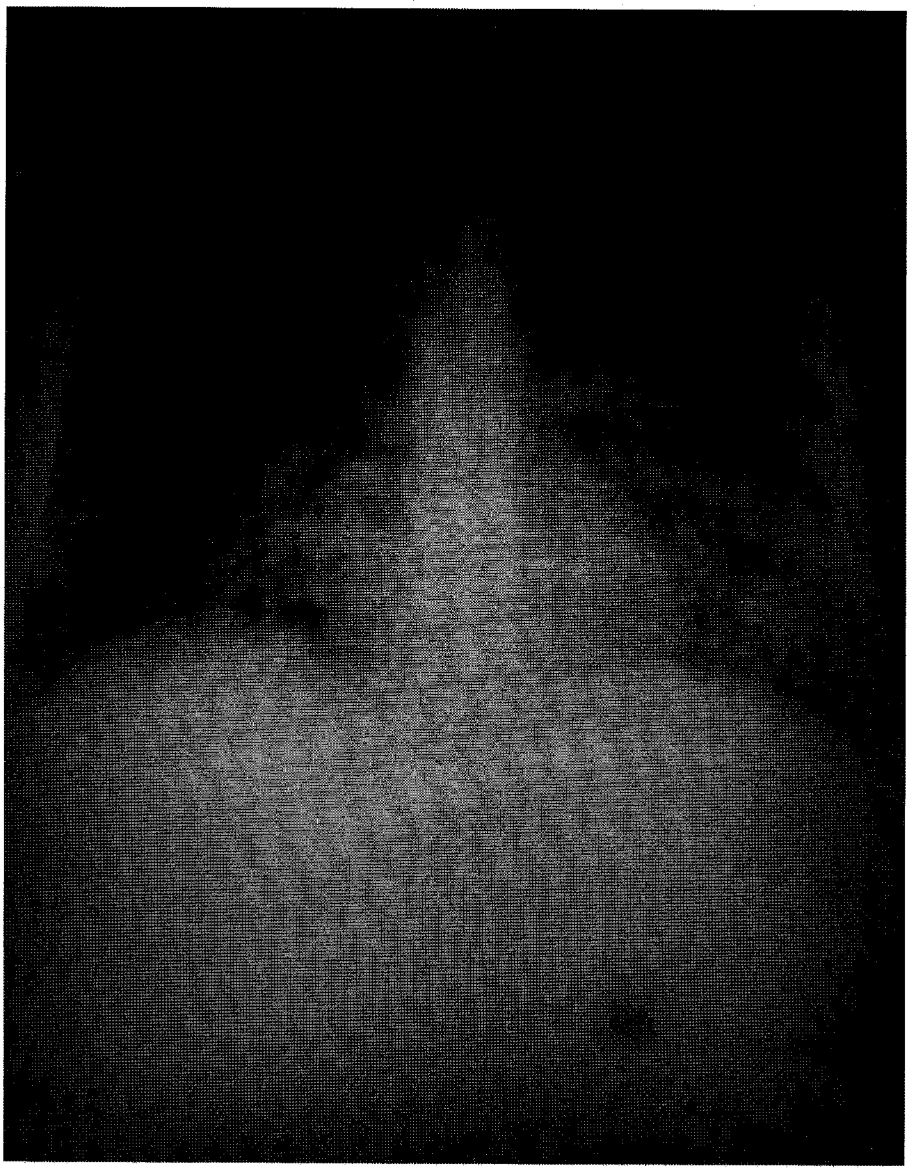

Fig. 1. Admission chest radiograph of case 2 demonstrating a severe, patchy, and diffuse alveolar infiltrate consistent with varicella pneumonia.

with diffuse pneumonitis or ARDS (Fig. 2). She was started on 100% oxygen by non-rebreather mask, acyclovir, and erythromycin. Within 24 h, her breathing became more labored requiring intubation and ventilatory support. On the 3rd hospital day, the patient developed disseminated intravascular coagulation (DIC). She was noted to have vaginal bleeding on this day and a cervical examination revealed 3 cm dilatation with bulging membranes. Given clinically worsening DIC, a prostin induction was begun to expedite delivery. She delivered a non-viable fetus 10 h later. Following delivery, the patient's condition further deteriorated necessitating inverse inspiratory-to-expiratory ventilatory support, invasive hemodynamic monitoring, vasopressor support, total parenteral nutrition, and multiple transfusions of blood components. Her hospital course was further complicated by ascites, cholecystitis with subsequent cholecystectomy, pneumothorax, tracheostomy secondary to prolonged ventilator dependence, and candidal pneumonitis requiring treatment with intravenous amphotericin B. She died 31 days after admission. Virologic studies and autopsy revealed that the patient expired with varicella pneumonia, ARDS, varicella hepatitis, and DIC.

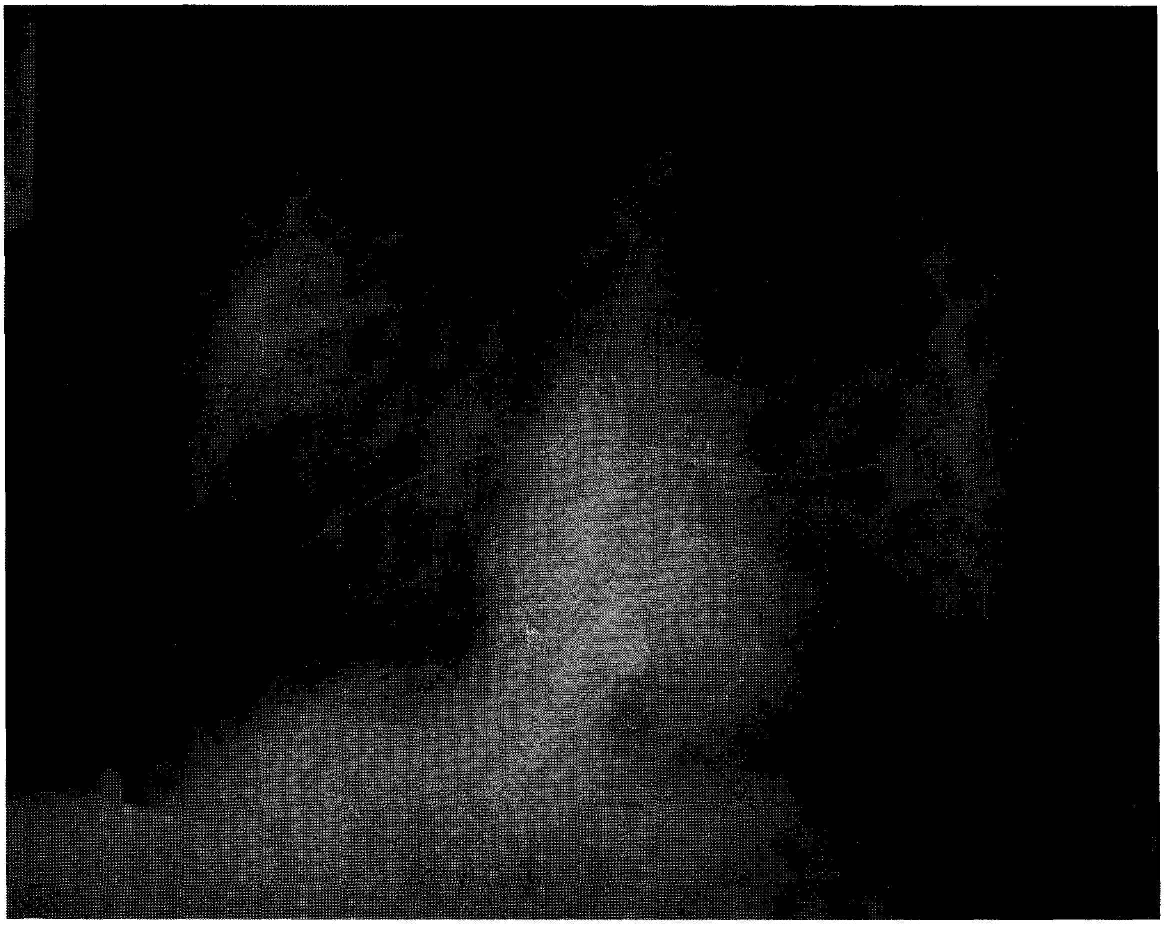

Fig. 2. Admission chest radiograph of case 3 demonstrating findings consistent with diffuse pneumonitis (ARDS).

### Case 4

T.K. was a 20-year-old white female gravida 1, para 0, at 30 4/7 weeks gestation who presented with a 2-day history of fever, malaise, myalgias, productive cough with increasing dyspnea, and a vesicular rash present over the face, trunk, and upper extremities. Her past medical history was significant for tobacco use and stage III Hodgkin's disease which had been in remission for 2 years prior to presentation. Initial physical examination revealed tachycardia, tachypnea, a temperature of 38.2°C, moderate amount of dyspnea, bilateral wheezing on auscultation, and diffuse vesicular lesions present on the face, trunk, and extremities. Chest X-ray revealed diffuse, bilateral coarse nodular pneumonia. The patient was started on intravenous acyclovir and cefuroxime. Despite albuterol aerosol treatments and aggressive pulmonary toilet, she became more hypoxic requiring intubation and ventilatory support. A repeat chest X-ray revealed pneumonia with evidence of ARDS. She was given varicella-zoster immune globulin (VZIG; 625 units intramuscularly) secondary to her immunocompromised status and probable preterm delivery. Preterm labor was confirmed, and she was started on intravenous magnesium sulfate for tocolysis. On hospital day 10, the patient began to appear septic, and was started on broad spectrum antibiotics. She was delivered of a normal female infant by primary low transverse cesarean section secondary to nonreassuring fetal assessment by non-stress test, biophysical profile, and contraction stress test. The infant had Apgar scores of 2 and 5 at 1 and 5 min, respectively, and a weight of 2,015 g. Her condition slowly improved, and she was extubated on postoperative day 9 and discharged from the hospital on postoperative day 12.

### Case 5

L.F. was a 32-year-old white female gravida 5, para 3, who presented at 27 5/7 weeks gestation with a

1-week history of dyspnea cough productive of green sputum. She related a 1-day history of a vesicular rash present on her trunk and face. She had recent exposure to varicella infection. The patient's past medical history was significant for asthma, chronic bronchitis, and insulin-requiring gestational diabetes mellitus. She denied any tobacco use. Initial physical examination revealed a temperature of 38.1°C, mild tachycardia, tachypnea, weight of 175.5 kg, mild respiratory distress, diffuse wheezing throughout both lung fields, and vesicular lesions scattered over her face, neck, and trunk. Chest X-ray revealed a diffuse interstitial process bibasilarly consistent with varicella pneumonia. Initial obstetric ultrasound revealed a viable singleton fetus with decreased amniotic fluid volume, but no apparent fetal anomalies. Varicella antibody studies, comprehensive viral studies, sputum culture, and acid-fast bacilli (AFB) studies were all negative. The patient was admitted and started on ceftriaxone, acyclovir, oral clarithromycin, subcutaneous heparin, and intravenous aminophylline. Her hospital course was unremarkable except for the development of thrombocytopenia, which was attributed to her viral syndrome after pregnancy-induced hypertension was excluded. At 32 weeks gestation, she was delivered by low vertical cesarean section secondary to decreased fetal movement, a positive contraction stress test, severe oligohydramnios, and the inability to effectively monitor the patient due to obesity. She was delivered of a normal male infant weighing 1,097 g, with Apgar scores of 5 and 9 at 1 and 5 min, respectively. The patient had an uncomplicated postpartum course.

### Case 6

L.H. was a 34-year-old female gravida 4, para 3, who presented at 30 weeks gestation with a 2-day history of pruritic rash followed by the development of fever, chills, dyspnea, and non-productive cough. Her past medical history was unremarkable except for exposure to varicella infection 2 weeks before presentation. She denied any history of tobacco use. Initial physical examination revealed a temperature of 39.4°C, mild tachycardia, mild tachypnea, and rales present at the posterior base of the left lung. She was noted to have an extensive maculopapular rash with lesions in various stages from pustules to scarred lesions involving the face, neck, trunk, abdomen, back, extremities, and oropharynx. Chest X-ray revealed a diffusely nodular interstitium with a left lower lobe infiltrate and small bilateral pleural effusions. Blood cultures, sputum cultures, varicella antibody studies, and comprehensive viral studies were all negative. The patient was treated with intravenous acyclovir and was discharged without complications. She subsequently presented at 38 weeks gestation to have an uncomplicated spontaneous vaginal delivery of a normal female infant weighing 4,275 g.

### Case 7

L.B. was a 18-year-old white primigravid female with an intrauterine pregnancy at 23 weeks gestation who presented with a history of chest congestion, vesicular skin rash, fever, productive cough, and progressively worsening shortness of breath. Her past medical history was unremarkable with the exception of exposure to varicella-zoster infection 2 weeks prior to the onset of her symptoms and tobacco use. Initial physical examination revealed a temperature of 37.6°C, tachycardia, mild tachypnea, and diffuse vesicular rash present on the face, trunk, extremities, abdomen, and back. Auscultation of the lungs revealed only fair air exchange with few bibasilar rales. Initial chest X-ray revealed bilateral patchy nodular infiltrates consistent with varicella pneumonia. An obstetric ultrasound revealed a viable female fetus with no evidence of fetal anomalies. The patient was admitted with a diagnosis of varicella pneumonia. She received acyclovir, oxygen (2 l by nasal cannula), VZIG, aerosol treatments, and cefazolin for a concomitant urinary tract infection. The patient recovered without complications. All viral, bacterial, and mycoplasma cultures were reported as negative. The patient returned at term to have an uncomplicated spontaneous vaginal delivery of a normal female infant weighing 3,033 g.

### DISCUSSION

Varicella, which is caused by a DNA virus, is usually a highly contagious benign childhood disease. In adults the illness is less common but usually more severe. Because varicella infection is highly contagious, it is estimated that only 5% of reproductive age women lack IgG antibody.[4] Only 2% of reported cases occur after the age of 20 years, but 25% of deaths from varicella are in this age group.[5]

The most common complication is varicella pneumonitis, occurring in 14–50% of adults with a mortality rate of 3–11%.[6,7]

The incidence of varicella infection in pregnancy is 1–7/10,000 pregnancies.[1,8] Varicella infection is considered to be more severe in pregnancy,[9] with a mortality from varicella pneumonia estimated to be as high as 44% in pregnant women compared to 11% in non-pregnant women.[3,4,6] The mortality from varicella-zoster pneumonia is highest if infection occurs in the third trimester. This increased risk of fatality is postulated to be secondary to advancing immmunosuppression during later pregnancy though this has never been proved.[2,10] In the cases presented, all women had infection in the second and third trimesters, with the only maternal death occuring in the early second trimester.

Fetal complications of varicella-zoster infection in pregnancy include premature delivery, fetal varicella syndrome, congenital zoster of infancy, and neonatal varicella.[3,11–14] Fetal varicella syndrome occurs when a fetus is exposed to varicella infection in the first trimester of pregnancy and is characterized by low birth weight, cutaneous scars, hypotrophic limbs, ocular abnormalities including chorioretinitis, cataracts, microphthalmia, and Horner's syndrome, brain damage, and mental retardation.[7,9] The exact incidence of this complication is unknown but has been estimated as 3–6% by prospective studies.[8,11,12] Neonatal varicella is most common and severe when maternal infection occurs within 4 days of delivery or 48 h following delivery.[7,9] This increased severity related to time of delivery is thought to be secondary to a lack of passive immunity from maternal antibodies.[9] Neonatal varicella infection is associated with complications including pneumonia, hepatitis, thrombocytopenia, arthritis, conjunctivitis, carditis, and nephritis, and is estimated to have a case fatality rate of 30–50%.[7,9] None of the neonates presented developed any evidence of varicella infection or fetal varicella syndrome.

Acyclovir is a nucleoside analogue that inhibits replication of herpes virus DNA synthesis.[15] Acyclovir is a category C medication and therefore should not be used in pregnancy unless the potential therapeutic benefits outweigh the potential risks to the fetus.[16] Acyclovir does cross the placenta and is found in amniotic fluid and fetal tissue, though no teratogenic effects have been observed in 241 pregnant women exposed over 6 years experience with this agent.[16] The first use of intravenous acyclovir in pregnancy for treatment of varicella pneumonia was reported in 1986.[17] Prior to the use of acyclovir for varicella pneumonia in pregnancy, the mortality was 36–41%.[3,4] A significant reduction in mortality to 13% has been reported since the advent of acyclovir therapy in the treatment of varicella pneumonia.[4] The exact dosages reported in studies have varied from 5 to 30 mg/kg.[17–22]. The dose usually recommended by infectious disease experts for treating disseminated varicella-zoster infections in immunocompromised patients is 10–15 mg/kg given 3 times daily for 7 days.[23–26] It should be noted that the one maternal death in our case series involved a patient who had initially received a low dose of acyclovir (6.5 mg/kg later increased to 10 mg/kg), though whether this contributed to her death is unclear.

VZIG is a product which contains varicella-zoster immunoglobulin G and is derived from the plasma of normal volunteer blood donors with high titers of the antibody.[5,27] VZIG has been available in the United States since 1978 and is commonly used successfully to treat infants exposed to varicella infection. It is also indicated in immunocompromised individuals and pregnant patients with no known history of infection and an unknown antibody status when exposed to varicella infection.[5] VZIG should be administered within 96 h of exposure to varicella to be effective.[27] When VZIG (adult dose 625 units intramuscularly) is administered to exposed adults, it has been shown to prevent or attenuate the course of infection,[27,28] but it has not been shown to prevent congenital or neonatal varicella.[5]

Varicella vaccine (Varivax) is a live attenuated vaccine which became available in the United States in 1995.[29] The recommended dosage of this vaccine is two intramuscular injections of 0.5 ml given 4–8 weeks apart.[29] In adults, two doses of vaccine are required to achieve a seroconversion rate of greater than 90%.[30] It has been proposed that limitations in adult helper T-cell response to varicella-zoster virus antigens are responsible for this observation.[31] The persistence of antibodies 10 years after vaccination is 95% in healthy children and 75% in adults.[32] Side effects from the vaccination occur in 5–10% of those vaccinated and usually consists of a mild rash.[32] Routine use of this vaccine will hopefully reduce the number of susceptible women entering pregnancy thereby decreasing the number of congenital/neonatal infections and the risk of significant morbidity and mortality associated with primary varicella infection in pregnancy. None of the patients presented in this report received the varicella vaccine.

In conclusion, primary maternal varicella-zoster infection in pregnancy is associated with significant morbidity and mortality as demonstrated by the cases presented in this report. Given the potential severity of this disease in pregnancy, routine antenatal screening for varicella antibodies and postpartum immunization similar to that of current rubella immunization practices should be considered. Theoretically, this practice would lower the risks of congenital varicella syndrome, neonatal herpes zoster, neonatal varicella infection, and maternal morbidity and mortality by decreasing the number of primary infections during pregnancy. The cost effectiveness of such an immunization program will need to be researched, but as evident from our case studies, the consequences of varicella-zoster infection in pregnancy can be devastating. The financial burden of caring for women with serious varicella infection in pregnancy also needs to be considered when evaluating the cost effectiveness and benefit of a routine screening and vaccination program.

### REFERENCES

1. Stagno S, Whitley RJ: Herpes virus infection in pregnancy. II. Herpes simplex and varicella infections. N Engl J Med 313:1327–1330, 1985.
2. Houston H, Sinnott JT, Murphy SJ, Larkin JA, Greene JN: Chickenpox in pregnancy. Infect Med 11:564–568, 1994.
3. Harris RE, Rhoades ER: Varicella pneumonia complicating pregnancy: Report of a case and review of literature. Obstet Gynecol 25:734–740, 1965.
4. Broussard RC, Payne K, George RB: Treatment with acyclovir of varicella pneumonia in pregnancy. Chest 99:1045–1047, 1991.
5. Centers for Disease Control: Varicella-zoster immune globulin for prevention of chickenpox: Recommendation of Immunization Practices Advisory Committee. Ann Intern Med 100:859–865, 1984.
6. Straus SE, et al.: Varicella-zoster virus infections: Biology, natural history, treatment, and prevention (NIH conference). Ann Intern Med 108:221–237, 1988.
7. Clark GPM, Dobson PM, Thickett A, Turner NM: Chickenpox pneumonia: Its complications and management. Anesthesia 46:337–380, 1991.
8. Balducci J, Rodis JF, Rosengren S, Vintzileos AM, Spivey G, Vosseller C: Pregnancy outcome following first trimester varicella infection. Obstet Gynecol 79:5–6, 1992.
9. Brunell PA: Varicella in pregnancy, the fetus, and the newborn: Problems in management. J Infect Dis 166:S542–547, 1992.
10. Smego RA, Asperilla MO: Use of acyclovir for varicella pneumonia during pregnancy. Obstet Gynecol 78:1112–1116, 1991.
11. Siegel M: Congenital malformation following chickenpox, measles, mumps, and hepatitis: Results of a cohort study. JAMA 226:1521–1524, 1973.
12. Paryani SG, Avin AM: Intrauterine infection with varicella-zoster virus after maternal varicella. N Engl J Med 314:1542–1546, 1986.
13. Pearson HE: Parturition varicella-zoster. Obstet Gynecol 23:21–27, 1964.
14. Brunnell PA: Varicella-zoster infections in pregnancy. JAMA 199:315–317, 1967.
15. Brown ZA, Baker DA: Acyclovir therapy during pregnancy. Obstet Gynecol 73:526–531, 1989.
16. Andrews EB, Yankaskas BC, Cordero JF, Schoeffler K, Hampp S: Acyclovir in pregnancy registry: Six years experience. Obstet Gynecol 79:7–13, 1992.
17. Landsberger EJ, Hager WD, Grossman JH III: Successful management of varicella pneumonia complicating pregnancy. J Reprod Med 31:311–314, 1986.
18. Glaser JB, Loftus J, Ferragamo V, Mootbar H, Castellano M: Varicella-zoster infection in pregnancy (letter). N Engl J Med 315:1416, 1986.
19. Hankins GD, Gilstrap LC, Patterson AR: Acyclovir treatment of varicella pneumonia in pregnancy (letter). Crit Care Med 15:336, 1987.
20. Leen CLS, Mandel BK, Ellis ME: Acyclovir and pregnancy (letter). Br Med J 295:308, 1987.
21. Eder SE, Apuzzio JJ, Weiss G: Varicella pneumonia during pregnancy: Treatment of two cases with acyclovir. Am J Perinatol 5:16–18, 1988.
22. Boyd K, Walker E: Use of acyclovir to treat chickenpox in pregnancy. Br Med J 296:393–394, 1988.
23. Dorsky DI, Crumpacker CS: Drugs five years later—Acyclovir. Ann Intern Med 107:859–874, 1987.
24. Drugs for viral infections. Med Lett Drugs Ther 32:73–78, 1990.
25. Balfour HH: Varicella-zoster virus infections in immunocompromised hosts: A review of the natural history and management. Am J Med 85(Suppl 2A):68–73, 1988.
26. Peterslund NA: Management of varicella-zoster infections in immunocompetent hosts. Am J Med 85(Suppl 2A):74–78, 1988.
27. Varicella-Zoster Immune Globulin (Human)—Package Insert.

28. Enders G: Management of varicella-zoster contact and infection in pregnancy using a standardized varicella-zoster ELISA test. Postgrad Med J 61:23–30, 1985.

29. Licensure of varicella virus vaccine, live. JAMA 273: 1253, 1995.

30. Gershon AA, Steinberg SP, LaRussa P, Ferrara A, Hammerschlag M, Gelb L, and NIAID Varicella Vaccine Collaborative Study Group: Immunization of healthy adults with live attenuated varicella vaccine. J Infect Dis 158:132–137, 1988.

31. Nader S, Bergen R, Sharp M, Arvin AM: Age-related differences in cell-mediated immunity to varicella-zoster virus among children and adults immunized with live attenuated varicella vaccine. J Infect Dis 171:13–17, 1995.

32. Gershon AA: Varicella vaccine. Isr J Med Sci 30:482–484, 1994.

## References

[PDF_00631] Andrews E. B., Yankaskas B. C., Cordero J. F., Schoeffler K., Hampp S. (1992). Acyclovir in pregnancy registry: six years' experience. The Acyclovir in Pregnancy Registry Advisory Committee.. Obstet Gynecol.

[PDF_00609] Balducci J., Rodis J. F., Rosengren S., Vintzileos A. M., Spivey G., Vosseller C. (1992). Pregnancy outcome following first-trimester varicella infection.. Obstet Gynecol.

[PDF_00654] Balfour H. H. (1988). Varicella zoster virus infections in immunocompromised hosts. A review of the natural history and management.. Am J Med.

[PDF_00648] Boyd K., Walker E. (1988). Use of acyclovir to treat chickenpox in pregnancy.. Br Med J (Clin Res Ed).

[PDF_00596] Broussard R. C., Payne D. K., George R. B. (1991). Treatment with acyclovir of varicella pneumonia in pregnancy.. Chest.

[PDF_00629] Brown Z. A., Baker D. A. (1989). Acyclovir therapy during pregnancy.. Obstet Gynecol.

[PDF_00627] Brunell P. A. (1967). Varicella-zoster infections in pregnancy.. JAMA.

[PDF_00650] Dorsky D. I., Crumpacker C. S. (1987). Drugs five years later: acyclovir.. Ann Intern Med.

[PDF_00645] Eder S. E., Apuzzio J. J., Weiss G. (1988). Varicella pneumonia during pregnancy. Treatment of two cases with acyclovir.. Am J Perinatol.

[PDF_00669] Gershon A. A., Steinberg S. P., LaRussa P., Ferrara A., Hammerschlag M., Gelb L. (1988). Immunization of healthy adults with live attenuated varicella vaccine.. J Infect Dis.

[PDF_00678] Gershon A. A. (1994). Varicella vaccine.. Isr J Med Sci.

[PDF_00593] HARRIS R. E., RHOADES E. R. (1965). VARICELLA PNEUMONIA COMPLICATING PREGNANCY. REPORT OF A CASE AND REVIEW OF LITERATURE.. Obstet Gynecol.

[PDF_00640] Hankins G. D., Gilstrap L. C., Patterson A. R. (1987). Acyclovir treatment of varicella pneumonia in pregnancy.. Crit Care Med.

[PDF_00634] Landsberger E. J., Hager W. D., Grossman J. H. (1986). Successful management of varicella pneumonia complicating pregnancy. A report of three cases.. J Reprod Med.

[PDF_00674] Nader S., Bergen R., Sharp M., Arvin A. M. (1995). Age-related differences in cell-mediated immunity to varicella-zoster virus among children and adults immunized with live attenuated varicella vaccine.. J Infect Dis.

[PDF_00625] PEARSON H. E. (1964). PARTURITION VARICELLA-ZOSTER.. Obstet Gynecol.

[PDF_00622] Paryani S. G., Arvin A. M. (1986). Intrauterine infection with varicella-zoster virus after maternal varicella.. N Engl J Med.

[PDF_00657] Peterslund N. A. (1988). Management of varicella zoster infections in immunocompetent hosts.. Am J Med.

[PDF_00619] Siegel M. (1973). Congenital malformations following chickenpox, measles, mumps, and hepatitis. Results of a cohort study.. JAMA.

[PDF_00616] Smego R. A., Asperilla M. O. (1991). Use of acyclovir for varicella pneumonia during pregnancy.. Obstet Gynecol.

[PDF_00587] Stagno S., Whitley R. J. (1985). Herpesvirus infections of pregnancy. Part II: Herpes simplex virus and varicella-zoster virus infections.. N Engl J Med.

[PDF_00603] Straus S. E., Ostrove J. M., Inchauspé G., Felser J. M., Freifeld A., Croen K. D., Sawyer M. H. (1988). NIH conference. Varicella-zoster virus infections. Biology, natural history, treatment, and prevention.. Ann Intern Med.

